# Unpacking multi-trophic herbivore-grass-endophyte interactions: feedbacks across different scales in vegetation responses to Soay sheep herbivory

**DOI:** 10.1007/s00114-018-1590-9

**Published:** 2018-11-20

**Authors:** Mark Vicari, Adriana Puentes, Gustaf Granath, Jennifer Georgeff, Fiona Strathdee, Dawn R. Bazely

**Affiliations:** 1 Department of Biology, York University, 4700 Keele St., Toronto, ON Canada; 2 Department of Ecology, Swedish University of Agricultural Sciences, Box 7044, 750 07 Uppsala, Sweden; 3 Department of Ecology and Genetics, Evolutionary Biology Centre, Uppsala University, Norbyvägen 18D, Uppsala, Sweden

**Keywords:** Aboveground net primary production (ANPP), *Epichloë festucae*, Grazing optimization, Herbivore irruptions, Inducible defenses, Plant demography

## Abstract

Grazing can induce changes in both plant productivity and nutritional quality, which may subsequently influence herbivore carrying capacity. While research on Soay sheep (*Ovis aries* L.) dynamics on Hirta Island in the St. Kilda archipelago has elucidated the complexity of population drivers, including parasites, the role of herbivore-generated feedbacks as an intrinsic regulating factor remains unclear. The sheep lack large predators and every 3–9 years undergo population crashes (overcompensatory mortality). We investigated the effects of grazing on (1) sward productivity and (2) quality (toxicity) of the primary forage species, red fescue (*Festuca rubra* L.), which is highly infected by an alkaloid-synthesizing fungal endophyte. Grazing had a negative impact on both forage quantity and quality. At higher sheep densities, impacts on sward growth were magnified, resulting in a nonlinear relationship with plant productivity. Simultaneously, endophyte hyphal load (and by inference, toxicity) peaked close to the time of a crash. A greenhouse experiment showed that alkaloid concentration in *F. rubra* increased in response to artificial defoliation. We conclude that at high sheep densities, grazing-mediated reductions in productivity, together with sustained alkaloid production, are likely to influence sheep dynamics. Future research should consider the interactive effects of forage toxicity, quantity, and nutritional content.

## Introduction

The question of how populations are regulated remains a central topic of ongoing debate and research in ecology (e.g., Krebs 2013; Wilkinson and Sherratt 2016; Letnic et al. 2018). For herbivores, the quantity and quality of available plant forage may have a strong influence in limiting population size when carnivores are absent or rare, resulting in bottom-up regulation (Schmitz 2008). However, even in the absence of predators, herbivore population regulation may be complex due to feedbacks between herbivores and plants. While high levels of herbivory may reduce plant productivity due to photosynthetic tissue removal, moderate herbivory may enhance productivity, through reduced self-shading or accelerated nutrient cycling (McNaughton 1979; Bazely and Jefferies 1989; Stewart et al. 2006).

Simultaneously, herbivores may experience reduced food quality by inducing host-plant chemical defenses (e.g., Reynolds et al. 2012). By altering plant productivity (van der Graaf et al. 2005) and/or food quality (Reynolds et al. 2012), herbivores can influence the carrying capacity of their environment, resulting in feedback regulation of their population. However, the effects of multiple feedbacks are rarely considered in combination (McNutt et al. 2017), despite the fact that they can have nonadditive effects on herbivore dynamics (Abbott et al. 2008).

When introduced to carnivore-free environments on islands or isolated mainland habitats, large mammalian herbivores typically show irruptive population growth in which an initial abundance of food leads to an overshoot of the habitat’s carrying capacity (reviewed in Gross et al. 2010). Overgrazing is followed by overcompensatory mortality (a population “crash”) and, in most instances, long-term changes to the vegetation community that lower the subsequent carrying capacity of the habitat. The ability of the vegetation to recover (and to sustain future irruptions) depends on its ability to tolerate and/or resist grazing, and thus varies with vegetation type (Kaeuffer et al. 2010; Ricca et al. 2014).

On the island of Hirta in the St. Kilda archipelago, Scotland, an introduced population of feral Soay sheep, *Ovis aries* L., exhibits periodic irruptions followed by overcompensatory mortality of up to 60% in a single season (Clutton-Brock et al. 1997; Grenfell et al. 1998). Crashes occur at intervals of 3 to 9 years (Hayward et al. 2014). Despite periodic heavy grazing, the island’s carrying capacity does not appear to be degrading over time, reflecting the resilience of its graminoid-based vegetation (Mysterud 2006). Carnivores are absent, and while parasites likely play a role in top-down regulation (Hayward et al. 2014), bottom-up effects are thought to be major determinants of sheep dynamics (Crawley et al. 2004). Garnier et al. (2017) showed that symptoms of starvation stress peak in conjunction with sheep numbers immediately prior to a crash, but are alleviated in sheep that survive the following year, pointing to food limitation as a trigger of the crashes. Weather patterns that affect plant productivity have been shown to influence sheep dynamics (Coulson et al. 2001), but the role of plant-herbivore feedbacks has not been explored in detail. Our study simultaneously examined the effects of herbivory on both the quantity and quality (toxicity) of forage in order to better understand how intrinsic plant-herbivore interactions contribute to sheep dynamics in this highly resilient irruptive system.

The grass red fescue, *Festuca rubra* L., is the sheep’s most preferred diet item (Crawley et al. 2004), being the largest single diet component (Milner and Gwynne 1974). The biomass or standing crop of *F. rubra* on Hirta is negatively correlated with sheep population density (Crawley et al. 2004). However, standing crop is not necessarily a good proxy for vegetation productivity, unless plant shoot and leaf turnover is known.

In the 1990s, microscopic fungal endophytes of temperate grasses, such as *Epichloë* sp., emerged as a significant management issue in the USA, due to the impact of the toxic alkaloids that they synthesize, on livestock (Cheplick and Faeth 2009). On Hirta, grazed *F. rubra* populations have high prevalence of the fungal endophyte *Epichloë festucae* Leuchtmann, Schardl & Siegel, and the frequency of infected grass shoots or tillers is positively correlated with local grazing pressure (Bazely et al. 1997). *F. rubra* tillers from closely grazed swards on Hirta had higher densities of endophyte hyphae in their leaves (*hyphal loads*; Puentes et al. 2007) compared with tillers from ungrazed tussocks (Bazely et al. 1997). Experimental defoliation of *F. rubra* from Hirta increased foliar concentrations of ergovaline (Bazely et al. 1997), an ergot alkaloid produced by the endophyte that is highly toxic to livestock (Clay and Schardl 2002). These responses suggest the endophyte could influence sheep dynamics via the production of inducible anti-herbivore defenses—i.e. defenses whose production is increased within a plant in response to herbivore damage (Karban and Baldwin 1997).

Our general hypothesis was that feedback regulation of the sheep population on Hirta, via grazing-mediated changes in food quantity and/or quality, influences the timing and magnitude of the sheep crashes. Our study tested two specific hypotheses:

1. *Plant productivity and relative growth rate (indicators of sheep forage availability) are negatively correlated with sheep density and grazing intensity, increasing the likelihood that the sheep population will periodically overshoot its carrying capacity.* This hypothesis stems from (a) the theoretical prediction and (b) empirical observation that moderate herbivory can stimulate plant productivity via an increase in relative growth rate (= overcompensation^1^) when light or nutrients are limiting to growth (McNaughton 1979; Hik and Jefferies 1990; Stewart et al. 2006). This in turn should support higher population growth rates of sheep at low to moderate sheep densities. However, higher herbivory levels at high densities may suppress plant productivity, either because growth rate is stimulated but not sufficiently to compensate for lost tissue (= partial compensation) or because there is no change (= no compensation) or a decrease (= damage) in growth rate (Belsky 1986). A drop in productivity at high sheep density would exacerbate the effects of competition, leading to higher mortality.
  We explored this question in field experiments over a 3-year period corresponding with high, low (following heavy mortality), and intermediate sheep densities, respectively. We used two different methods: (a) an ecosystem approach, where we measured the effects of grazing and nutrient addition (N and P) on total sward aboveground net primary production (ANPP) and live standing crop, and (b) a plant-level approach, in which we measured the effects of grazing on production of individual plant modules (tillers or rosettes) of three common species in the grass sward (Jewell et al. 1974). The first approach provided a measure of total plant biomass available to consumers, while the second gave insight into the mechanisms underlying herbivore-mediated changes in plant productivity.
2. *Endophyte-mediated toxicity of F. rubra increases with increasing herbivore numbers and grazing intensity.* Higher sheep densities were assumed to correspond with more frequent grazing of individual tillers. While simulated herbivory in the form of a single cutting increased ergovaline content of St. Kilda *F. rubra* (Bazely et al. 1997), the impact of repeated defoliation on ergovaline content is unknown. We examined the subplant effects of grazing by examining the effects of (a) Soay sheep grazing on the hyphal load of *F. rubra* in situ over the same 3-year period and (b) simulated grazing (cutting) at a range of different frequencies on ergovaline content of *F. rubra* under greenhouse conditions.

## Methods

### Study site

**Fig. 1:**
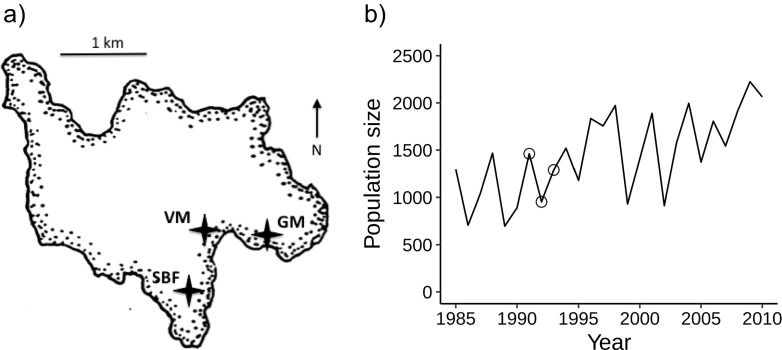
**a** The island Hirta, showing the locations of the St. Brianan Fanks (SBF), Village Meadows (VM), and Gun Meadow (GM). **b** Total sheep numbers on Hirta, 1985–2010. Circles = counts during study years 1991–1993 (modified from “St Kilda Soay Sheep Project,” http://soaysheep.biology.ed.ac.uk/population-ecology, accessed Aug 21 2018)

The St. Kilda archipelago (57° 50′ N, 8° 38′ W) is located 180 km off the west coast of Scotland. Hirta is the largest island (637 ha; Fig. 1a). Its grassland communities are dominated by *Agrostis* spp., *F. rubra*, *Holcus lanatus* L., and *Poa trivialis* L., and are grazed by feral Soay sheep (Jewell et al. 1974). Our study sites were at three major locations on Hirta (Fig. 1a): (1) St. Brianan Fanks (SBF), (2) western meadows of Village Bay (Village Meadows, VM), and (3) Gun Meadow (GM). The VM site lies on previously cultivated ground and is characterized by species-rich *Agrostis-Festuca* grassland (Jewell et al. 1974). SBF and GM are species-poor grassland with a high frequency of *F. rubra* growing on thin soils, and have never been cultivated.

Historically, population crashes of the Soay sheep occur in winters, following a summer in which the sheep population reaches or exceeds roughly 1200 animals (Clutton-Brock et al. 1997; Fig. 1b). In recent years, this carrying capacity number has increased as sheep body size has decreased—an effect attributed to climate change (Simmonds and Coulson 2015). Below carrying capacity, mortality is low and the population tends to increase, with lambing in the spring.

The field component of our study was conducted over three summers: 1991 to 1993. In the summer of 1991, the sheep population was 1449 animals. A crash occurred in February–March of 1992. In the summer of 1992, the population had declined by 35% compared with the previous year, to 957 animals. In 1993, the population increased to 1283 (Fig. 1b).

### ANPP

Six permanent, large exclosures made of cattle fencing (10 cm mesh) and wood were established in April 1991: three at VM (5 m × 5 m) and three at SBF (4 m × 4 m). Grass sward productivity in the absence of sheep grazing was estimated by sampling the vegetation inside these exclosures every 10–14 days, from late April to early October. On each sample date, nine 10 × 10-cm turves were randomly removed (destructive sampling), two from within each exclosure at VM (*n* = 6 total) and one from each at SBF (*n* = 3). All aboveground vegetation was cut at ground level from turves, sorted, washed, dried (60 °C for 72 h), and weighed. Standing crop, productivity, and *relative growth rate* (RGR; see below) for the combined live vascular component are reported here. ANPP for each 10 to 14-day interval was calculated by subtracting the mean live biomass at the beginning of the interval from that at the end. Cumulative ANPP for the season was calculated by adding ANPP for each interval.

Grass sward productivity in the presence of grazing was estimated using the portable exclosure method of Cargill and Jefferies (1984) to determine how much biomass grew immediately after protection from grazing over a short (10 to 14-day) interval. Two 50 × 50-cm portable chicken wire exclosures (3 cm mesh) were placed on the grazed sward in random locations within the 1–2-m strip around each large exclosure at VM (total = 6 portable exclosures). One portable exclosure was placed in each strip around each large exclosure at SBF (total = 3 portable exclosures). A 10 × 10-cm turf was sampled from within each portable exclosure at the beginning and end of each 10 to 14-day interval. Portable exclosures were moved to a new grazed location in the vicinity of each large exclosure on the first day of the next turf sampling interval. Consecutive sampling intervals in these grazed areas outside of the large exclosures ran throughout spring, summer, and autumn in 1991, 1992, and 1993 with some annual adjustments.

Plant productivity of grazed swards was measured at both VM and SBF in 1991 and 1992, and only at VM in 1993, using the same permanent large exclosures. Due to damage, one large VM exclosure was excluded in the 1993 sampling. The large SBF exclosures were storm-damaged during the 1992–1993 winter and could not be used.

Data collected for productivity measurements were used to calculate RGR for each 10 to 14-day sampling interval, using the formula RGR (g g^−1^ day^−1^) = [ln (*W*_*t*(*n* + 1)_) − ln (*W*_*t*(*n*)_)](*t*_(*n* + 1)_ − *t*_(*n*)_)^−1^ where *W* is the mean dry plant biomass per square meter for the grazed or ungrazed sward at each exclosure and *t*(*n*) is the day of the season on which the interval *n* began (Hunt 1982). An average seasonal RGR for grazed or ungrazed vegetation was then calculated by taking the mean of all intervals.

### Nutrient limitations to biomass growth

In 1992, a field experiment was carried out to determine if nutrient addition to vegetation (as may occur under grazing) would stimulate sward growth. We measured the effects of nitrogen (NH_4_NO_3_) and phosphorus (NaPO_4_) addition, separately and in combination, on plant growth and biomass in exclosed vegetation protected from sheep grazing.

Three chicken-wire exclosures—4 × 4 m at the base and 1 m tall—were built: two at VM and one at SBF. Each exclosure was subdivided into 50 × 50-cm rows and columns with string, creating a 4 × 4 set of subplots in which each row and column was separated by a 50-cm-wide pathway.

A Latin square plot design was used. Every 12 days from May to August 1992, 7.64 g of NH_4_NO_3_ or 8.05 g of NaPO_4_ was applied to each 50 cm × 50 cm subplot, according to the assigned treatment combination: control subplots received no salts, N subplots received only NH_4_NO_3_, P subplots received only NaPO_4_, and N + P subplots received both (*n* = 4 in each case). The live and dead biomass in each subplot was sampled by removing a 10 × 10-cm turf at the beginning, middle, and end of the experiment, as well as removing, washing, and sorting vegetation to graminoids, dicots, moss, and dead matter fractions.

### Leaf demography of three forage species: *F. rubra*, *Ranunculus acris*, and *Leontodon autumnalis*

#### Graminoids*: Festuca rubra*

Three pairs of 50 × 50-cm plots were established in each of VM, SBF, and GM on April 30–May 1, 1991 (*n* = 18 plots in 9 pairs). One plot of each pair was protected from grazing using a chicken-wire exclosure; the other (not more than 50 cm away) served as the grazed control. Within each plot, 36 tillers (arranged in a 40 × 40-cm grid with 6 tillers per row spaced at 8-cm intervals) were ringed around the base using colored electrical wire placed so that it was flush with the ground. On May 11–15, the leaves of each tiller were marked with waterproof ink at the base of each blade using a repeating sequence of one, two, or three dots, beginning with the oldest leaf (Bazely and Jefferies 1989). Leaves were recorded as emerging (if < 5 mm), live, senescent or dead, and grazed where appropriate. Leaves were re-assessed, and new leaves marked, every 10–24 days (for a total of 12 scorings) between May 23 and Oct. 1. The number of live leaves per tiller (i.e., standing crop of live leaves) was observed and recorded on each scoring date. The cumulative number of leaves, cumulative leaf deaths, and lifespan of the first two leaves produced after May 11 were calculated for each tiller. Only tillers that survived the entire season were included in the analysis.

#### Dicotyledonous species: *R. acris* and *L. autumnalis*

We monitored leaf turnover in *Ranunculus acris* L. and *Leontodon autumnalis* L., two relatively abundant and frequently consumed forbs (personal observation). Eight 50 × 50-cm plot pairs, spaced 5–10 m apart, were established at VM in June 1993. Within each plot, four randomly selected shoots of *R. acris* and *L. autumnalis* were ringed around the base with colored wire. None of these wires was dislodged during the season; thus, data were collected for all 128 plants. Leaf births and/or deaths were recorded every 11–26 days from June 16 until September 6, 1993 (5 scorings total). For *R. acris*, the first leaf produced after June 16 was used to calculate leaf lifespan. For *L. autumnalis*, only cumulative leaf births (not deaths) were recorded. Leaf length was recorded for both species. For *L. autumnalis*, the length of only the 5th-youngest leaf (the youngest fully expanded leaf) was recorded. Bud production in *L. autumnalis* (a nonrhizomatous species) was recorded.

### Hyphal load and ergovaline content of *F. rubra*

#### Hyphal load in *F. rubra* plants from in situ forage samples

Koh et al. (2006) found that endophyte-detection kits using monoclonal antibodies were accurate for dried grass samples in long-term storage. Phytoscreen field tiller endophyte detection kits (Agrinostics Inc., Watkinsville, GA) developed in the late 1990s successfully identified which *F. rubra* tillers contained the fungal endophyte after the tillers had been oven-dried at 70 °C and stored at room temperature for 12 years. The kits have some limitations (Jensen et al. 2011) which do not apply to our species (Koh et al. 2006).

In 2005, we estimated hyphal loads of fungal endophytes in stored St. Kilda *F. rubra* grass tiller samples from the forage productivity experiments of 1991 (*n* = 6), 1992 (*n* = 6), and 1993 (*n* = 3). It was not possible to make microscopic hyphal counts from dried tiller samples, so we followed the quantitative method of Koh et al. (2006), which uses the intensity of tissue print color (red-green difference) on scanned immunoblot cards to estimate the density of fungal hyphae within grass leaf tissue.

#### Greenhouse experiment

We examined the effect of cutting frequency, as a proxy for grazing intensity, on ergovaline content of endophyte-infected (E+) *F. rubra.* We used five genotypes grown from seed collected on Hirta (from widely spaced (> 10 m), putatively different maternal genets) in 1991.

In mid-October 1998, a single tiller of each genotype was planted in a 4-in. 1.5-L plastic pot (*n* = 5 replicates) containing autoclaved pro-mix potting soil. Plants were randomized and grown in a heated greenhouse (25 °C) for 6 weeks with supplementary lighting (14 h light:10 h dark). The pots were rotated once per week. Beginning Jan 1, 1999, one pot per genotype was assigned to each of five cutting treatments: no cutting, one cut (after 4 weeks), two cuts (0 and 4 weeks), four cuts (0, 2, 4, and 6 weeks), and eight cuts (0, 1, 2, 3, 4, 5, 6, and 7 weeks). The plant in each pot was cut to a height of 7.5 cm. All plants were cut after 8 weeks. Immediately after each cut, the grass clippings were collected and stored at − 20 °C. At the end of the experiment, pooled clippings from each treatment pot were freeze-dried and sent to the Veterinary Diagnostic Laboratory in Corvallis, OR, USA, for quantification of their ergovaline content.

### Statistical analyses

To examine the effects of grazing on total sward aboveground net primary production (ANPP) and live standing crop, mixed models were fitted. Plot was treated as a random factor nested within a block. Block (also random) represented the location where paired grazed and ungrazed plots were situated. Site (VM and SBF) and grazing (ungrazed (within exclosure) and grazed (outside exclosure)) were modeled as fixed effects, including all interactions. To determine if seasonal response patterns differed between grazing treatments, we included time (days since the first sampling date) as a fixed continuous variable with a second-degree polynomial, and its interaction with grazing. For the productivity data, we also analyzed the last sampling point in each season (i.e., no within-year repeated measurement) and examined the effect of grazing, location, year, and their interactions. The year effect and possible interactions were tested for standing crop by fitting a model without the time variable to pool over the whole season, with year as a fixed factor. Residual analyses indicated that the data violated the requirement for homogeneity of variance. To avoid transformation of the response variable, we used an exponential variance structure (*varExp* in the R package *nlme*) to model the increasing spread of residuals (Zuur et al. 2009).

To test the effects of N and P on plant growth, we performed ANOVAs on standing crop at the end of the season (after 3 months of fertilizer treatment).

The effect of grazing in the field on plant level responses (leaf lifespan, leaf births, leaf deaths, and leaf length) was analyzed by mixed-model ANOVAs treating site (VM, SBF, and GM) and grazing as fixed factors and block as a random factor. Time since first sampling was included as an ordered factor to examine the response patterns over the experimental period.

For the in situ hyphal load analysis, some of the plot samples were of insufficient quality for endophyte detection, or endophytes were not present in the specific tiller tested. Thus, we pooled measurements from the same plot over specific time periods of interest: before the crash (August–September 1991), just after the crash (May–June 1992), late 1992 (August–September 1992), and fall 1993 (August–September 1993) when the population had started to recover. To investigate if grazing had an impact on hyphal load and if this could be associated with sheep population fluctuations, we performed a Student’s *t* test comparing grazed and ungrazed plots at each time period. If needed, the response variable (red-green coloration) was log transformed to achieve homogeneity of residuals. Ergovaline levels in greenhouse plants were analyzed as a mixed-model ANOVA with genet as a random factor and number of cuts as an ordered categorical fixed factor.

Statistical analyses were performed using R Statistical Software version 3.0 (R core team 2013). Mixed models were analyzed using the R package *nlme* ver 3.1-109 (Pinheiro et al. 2013).

## Results

### Ecosystem responses

#### Plant standing crop and productivity

**Fig. 2:**
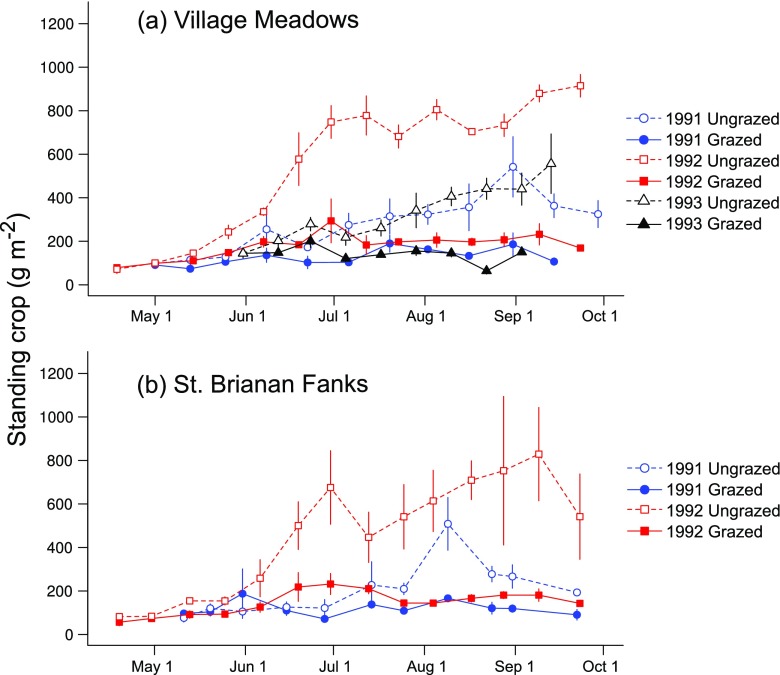
Standing crop of living biomass (dry weight, excluding bryophytes) in ungrazed (open symbols) and grazed (closed symbols) plots in **a** the Village Meadows and **b** the St. Brianan Fanks. Circles = 1991; squares = 1992; triangles = 1993. Mean ± SE

Soay sheep grazing reduced live standing crop in both VM and SBF localities in all years (Grazing, 1991, *F*_1,4_ = 19.0, *P* = 0.01; 1992, *F*_1,4_ = 103.0, *P* = 0.0005; 1993, *F*_1,3_ = 38.3, *P* = 0.01). Standing crop in grazed plots was relatively stable over the season. In ungrazed plots, biomass increased initially, and then leveled out (time × grazing; 1991, *F*_2,115_ = 9.6, *P* = 0.0001; 1992, *F*_2,152_ = 16.9, *P* = 0.0001; 1993, *F*_2,66_ = 39.2, *P* < 0.0001). The patterns were similar for VM and SBF in all years (Fig. 2).

**Fig. 3:**
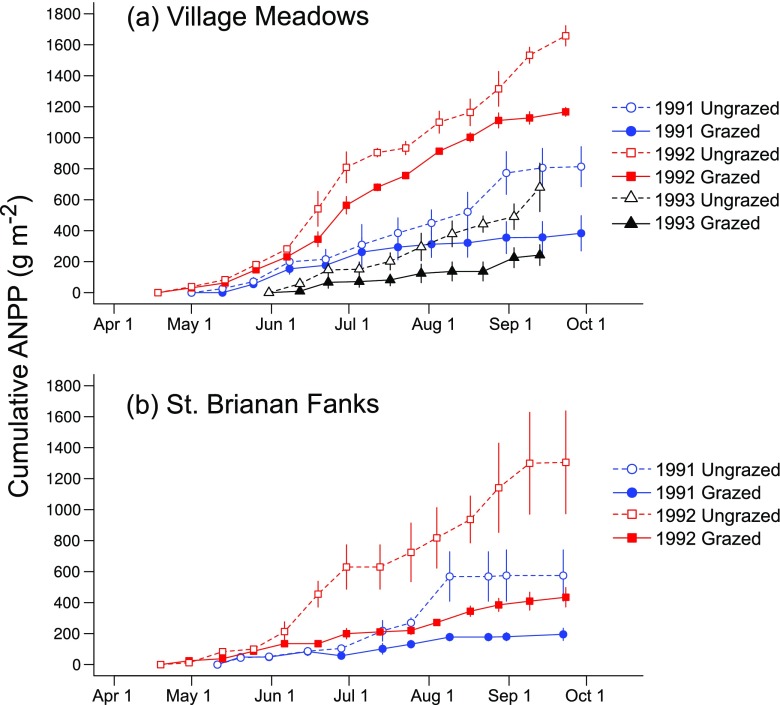
Cumulative aboveground net primary production in ungrazed (open symbols) and grazed (closed symbols) plots over the growing season in **a** Village Meadows and **b** the St. Brianan Fanks. Circles = 1991; squares = 1992; triangles = 1993. Mean ± SE

Soay sheep grazing reduced cumulative seasonal ANPP in all years of the study at both VM (1991, 391 g m^−2^ (48% reduction); 1992, 491 g m^−2^ (30% reduction); 1993, 437 g m^−2^ (64% reduction)) and SBF (1991, 379 g m^−2^ (66% reduction); 1992, 871 g m^−2^ (67% reduction)). Cumulative ANPP in grazed plots increased slowly over the season, while in ungrazed plots, it increased rapidly, especially towards the end of the growing season (time × grazing; 1991, *F*_2,112_ = 22.6, *P* < 0.0001; 1992, *F*_2,142_ = 24.2, *P* < 0.0001; 1993, *F*_2,48_ = 12.2, *P* = 0.0001; Fig. 3). ANPP was greater overall, in 1992 than in both 1991 and 1993 (*F*_2,10_ = 11.2, *P* = 0.003).

The main effect of grazing between years was that vegetation standing crop was greater overall in 1992 (low sheep density) than in 1991 (high sheep density). The increase was five times larger in grazed compared with ungrazed plots (year × grazing, *F*_1,8_ = 19.4, *P* = 0.002). In 1993 (intermediate sheep density), only VM plots were measured, and standing crop was lower than in 1992, with grazed plots showing the largest drop (year × grazing, *F*_2,7_ = 7.7 *P* = 0.02). No significant interaction effect with site was found.

**Fig. 4:**
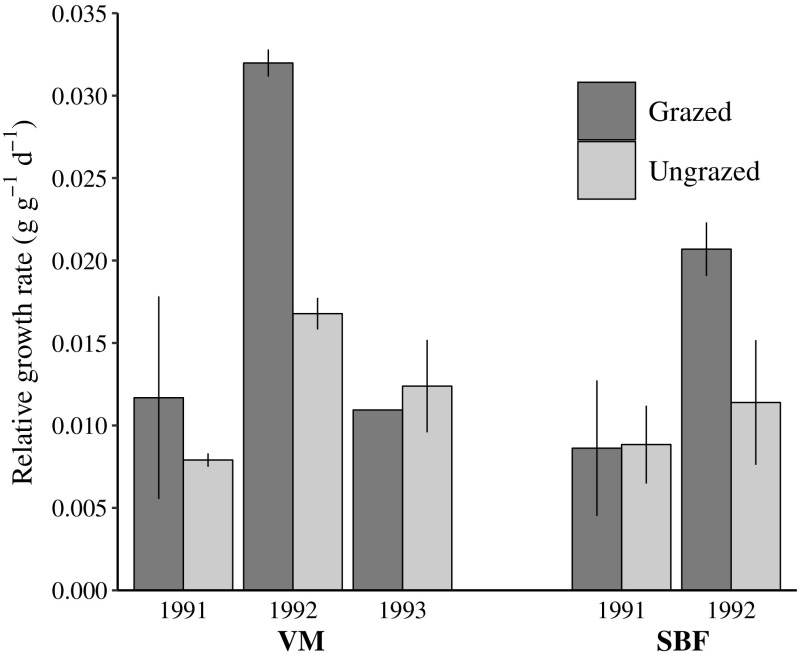
Relative growth rate (RGR, grams of dry weight produced per gram of dry plant tissue per day) of the swards in VM and SBF. Mean ± SE

There was an interaction effect between year and grazing in mean seasonal RGR (*F*_1,11_ = 5.0, *P* = 0.03). Grazed plots had higher RGR in 1992 than in 1991 or (in VM) 1993, but RGR in ungrazed plots varied little between years (Fig. 4). This pattern was consistent at both VM and SBF (year × grazing × site, *F*_1,8_ = 0.5, *P* = 0.82). RGR was stimulated by moderate grazing at low sheep density (1992; Fig. 4), but due to an accompanying reduction in standing crop, the stimulation was only large enough to achieve partial compensation in ANPP.

#### Nutrient application field experiment

**Fig. 5:**
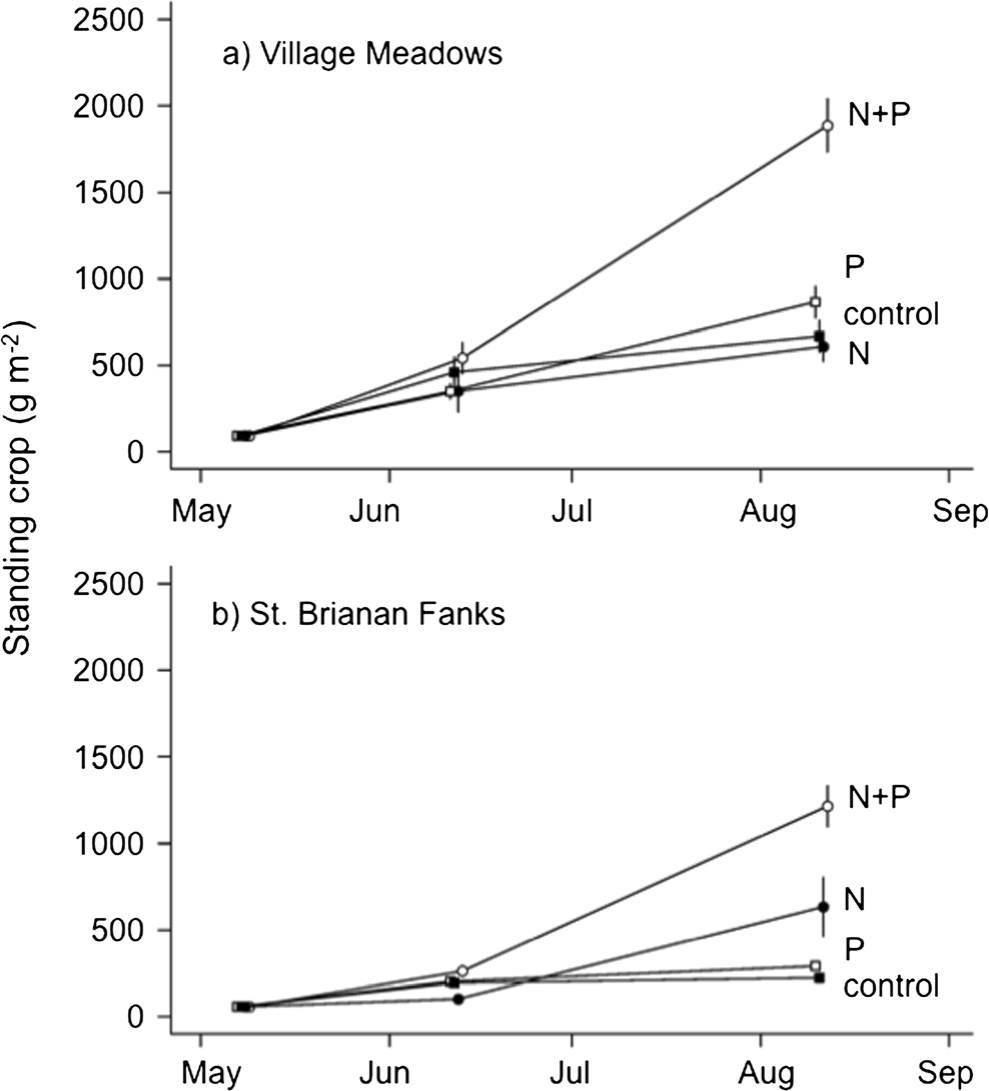
Standing crop (dry weight) of living biomass (excluding bryophytes) in exclosed swards amended with nitrogen (shaded circles), phosphorus (open squares), nitrogen and phosphorus in combination (open circles), or with no salts (shaded squares; control) in **a** Village Meadows and **b** St. Brianan Fanks. The N + P treatment was statistically different from the control at both sites, and N had a significant effect only at St. Brianan Fanks. Mean ± SE

Grass swards on Hirta required combined N + P additions to generate large effects on plant biomass at both VM and SBF (N × P, *F*_1,24_ = 12.0, *P* = < 0.0002; Fig. 5). However, we found a N × Site interaction (*F*_1,24_ = 18.1, *P* = 0.0002), with a positive effect of N addition on plant biomass at SBF (though much less than N + P additions) but no effect at VM. Phosphorus had a positive effect on plant biomass (P, F_1,24_ = 39.8, *P* < 0.0001), but the biomass change was small compared to the N × P treatment (Fig. 5).

Overall, VM swards had higher standing crop than SBF after 3 months (site, *F*_1,24_ = 46.7, *P* = < 0.0001). There was no significant N × P × site interaction (*F*_1,24_ = 1.0, *P* = 0.32).

### Plant level responses

#### Leaf demography of *F. rubra*

Grazing accelerated leaf production at all three study locations. Ungrazed tillers produced 7.1 ± 0.23 leaves (mean ± SE, *n* = 9 plots, with 18–32 plants per plot), while grazed tillers produced 8.9 ± 0.2 leaves (*n* = 9 plots, averaging 18–31 plants per plot) over the 151-day study period—a 25% increase (*F*_1,6_ = 158.2, *P* < 0.0001). The cumulative number of leaf deaths was 6.6 ± 0.2 for ungrazed, and 8.4 ± 0.4 for grazed, tillers (*F*_1,6_ = 43.8, *P* < 0.0006). Site had no effect on cumulative births or deaths. Grazing reduced leaf lifespan from 74.9 ± 2.7 days on ungrazed tillers to 63.2 ± 3.7 days on grazed tillers (*F*_1,6_ = 14.4, *P* = 0.009). Within grazed plots, both undamaged and damaged leaves had similarly short lifespans (64.8 ± 4.6 days and 60.5 ± 2.9 days, respectively).

**Fig. 6:**
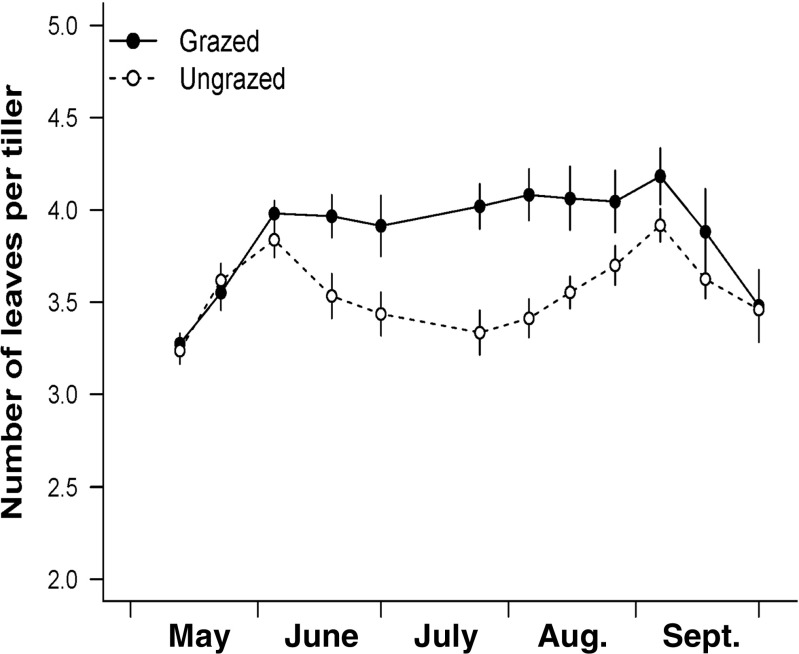
Number (standing crop) of living *F. rubra* leaves per tiller in grazed (closed circles) and ungrazed (open circles) field plots in the summer of 1991. Mean ± SE

The mean number of living leaves per tiller, averaged over the whole season, was slightly higher for grazed (3.9) than for ungrazed (3.6) tillers (*F*_1,5_ = 11.4, *P =* 0.02). However, there was a significant interaction effect between date and grazing treatment (*F*_11,110_ = 3.2, *P* = 0.0008; Fig. 6). From June 5 to July 25, the number of live leaves decreased on ungrazed tillers (due to an increase in death rate) while remaining more or less constant on grazed tillers. This suggests that older leaves in ungrazed plots were experiencing light limitation (Johnson and Parsons 1985).

#### Leaf demography of *R. acris* and *L. autumnalis*

Grazed shoots of *R. acris* and *L. autumnalis* produced 35% and 40% more leaves than ungrazed shoots, respectively (Table 1). Grazing reduced leaf size in both species (Table 1). In *R. acris*, petioles on grazed plants were 51% of the length of those on ungrazed plants, but blade lengths were similar. In *L. autumnalis*, leaves on grazed plants were 59% as long as those on ungrazed plants.

**Table 1:** Effects of grazing on demographic variables of dicot species *R. acris* and *L. autumnalis* analyzed with paired *t* tests. Data are means ± SE. *n* = 8

	*R. acris*			*L. autumnalis*		
	Grazed	Ungrazed	*P*	Grazed	Ungrazed	*P*
Cumulative leaves	4.2 ± 0.3	3.1 ± 0.4	0.006	14.8 ± 1.4	11.3 ± 1.0	0.004
Leaf length (mm)				66.5 ± 3.4	113.2 ± 8.7	< 0.001
Petiole	38.8 ± 5.5	75.8 ± 13.1	0.02			
Blade	13.3 ± 0.9	14.4 ± 1.2	0.18			

### Subplant response: endophyte interactions

#### Hyphal load in the field: 1991–1993

**Fig. 7:**
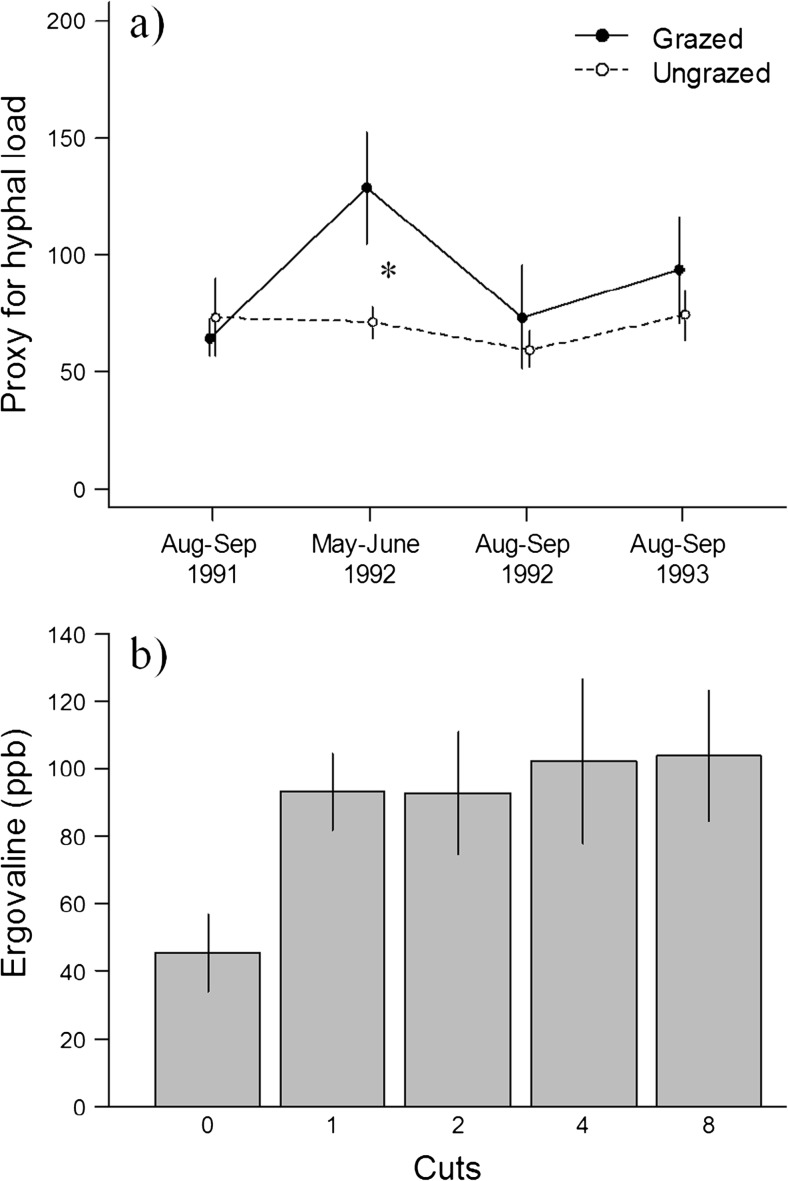
**a** Hyphal load as indicated by the intensity of green coloration on Phytoscreen Field Tiller Endophyte Detection Kit immunoblot cards (Agrinostics, Watkinsville, GA) for grazed (closed circles) and ungrazed (open circles) areas at different time periods. Asterisk represents a significant difference (*P* = 0.03) between the two treatments at a given period. Note that the periods are not equally separated in time. Mean ± SE. **b** Effect of cutting frequency on ergovaline concentrations in greenhouse-grown *F. rubra*. Mean ± SE. All treatments were significantly different from the control treatment (0 cuts)

The greatest estimated endophyte hyphal load was found in grazed tillers in the spring of 1992, immediately following the winter population crash (Fig. 7a): the effect of grazing on hyphal load was positive (*P* = 0.05). Grazing had no significant effect on hyphal load at any other sampling time.

#### Greenhouse experiment

Cutting E+ *F. rubra* tillers increased ergovaline levels (*F*_4,14_ = 6.1, *P* = 0.005; Fig. 7b), but this increase was only significant between uncut and cut tillers; amongst the four treatments involving one or more cuts, the frequency of cutting had no significant effect on ergovaline content.

## Discussion

Through a range of experiments, we explored whether intrinsic feedbacks between sheep numbers and the quantity or quality of the sheep’s forage exist that could influence the timing and magnitude of the sheep crashes. We found strong support for feedback with forage quantity and tentative support for feedback with forage quality.

### Hypothesis 1: Sheep density influences ANPP

Plant productivity and relative growth rate were negatively correlated with sheep density and grazing intensity, supporting hypothesis 1. While grazing suppressed ANPP at all sheep densities, RGR of the grazed sward was inversely related to sheep density. Thus, there was disproportionately lower ANPP at high sheep density, resulting in a larger decrease in the amount of food available to each individual sheep than would be the case if competition was the sole determinant of food availability. This suggests that feedback regulation of the sheep population can occur via grazing-mediated changes in ANPP/RGR that support rapid population growth at low sheep densities but worsen starvation (Garnier et al. 2017) at high densities, potentially causing a crash before overgrazing occurs. While other studies have documented changes in plant community composition and/or biomass during irruptions by large herbivores (Mysterud 2006; Gross et al. 2010; Starns et al. 2015), our study is the first to describe ANPP and RGR dynamics in a resilient, graminoid-based irruptive system.

Grazing accelerated leaf production in all three forage plant species studied, in line with other studies reporting an acceleration in leaf module turnover under grazing (e.g., Bazely and Jefferies 1989). While this has been identified as a mechanism by which grazing can increase productivity (Cargill and Jefferies 1984), on Hirta, increased leaf turnover was not associated with an increase in ANPP, likely due to an accompanying reduction in leaf size. The consistently negative effect of grazing on ANPP occurred despite the ungrazed sward sometimes being both light and (at SBF) nitrogen limited. Thus, any resources (light or nutrients) made available by grazing were not sufficient to enable the sward to fully compensate for plant tissue loss. The impact of grazing on root dynamics, which can influence the ability of plants to exploit nutrients mobilized by grazing (Fynn et al. 2017), merits future investigation in the St. Kilda system.

A single species can compensate for lost tissue in different ways depending on site and herbivore. On Hirta, we found that grazing of *F. rubra* decreased the lifespan longevity of leaves, including individual leaves that were not grazed. In contrast, van der Graaf et al. (2005) reported that grazing by barnacle geese, *Branta leucopsis* Bechstein, on an island in the Dutch Wadden Sea increased the longevity of existing leaves of *F. rubra*, and had no effect on leaf production by individual shoots.

### Hypothesis 2: Endophyte-mediated toxicity of *F. rubra* increases with increasing herbivore numbers and grazing intensity

This hypothesis was tentatively supported by our observation that hyphal loads in *F. rubra* tracked sheep density. The highest hyphal loads occurred in grazed plots in May–June 1992 (the earliest sample from that year), approximately 2 months after the winter sheep population crash ended. Hyphal loads in ungrazed plots were comparatively static. These results are in line with earlier findings (Bazely et al. 1997) and a recent common garden study (Fuchs et al. 2017) reporting a positive correlation between defoliation intensity and hyphal load.

In our greenhouse experiment, foliar ergovaline concentrations responded to defoliation with a roughly twofold increase, confirming an earlier finding (Bazely et al. 1997) that the alkaloid is inducible in E+ *F. rubra* from Hirta. However, we found the magnitude of induction was unaffected by cutting frequency. While this result does not support our hypothesis that ergovaline content of the sward increases with increasing sheep density, a role for forage toxicity cannot be discounted. More frequent hyphal load measurements over the course of a sheep crash cycle, coupled with in situ measures of alkaloid levels, will clarify the relationship between hyphal loading, ergovaline levels, and sheep dynamics.

Both our in situ and greenhouse experiments suggest that ergovaline content is maintained at high cutting frequencies. This contrasts with the findings of Belesky and Hill (1997) who showed that total ergot alkaloid concentration in E+ greenhouse-grown tall fescue, *Schedonorus phoenix* (Scop.) Holub, was lower in cut plants than in uncut controls, and was correlated with nonstructural carbohydrate concentration. Salminen and Grewal (2002) found that frequent mowing reduced ergot alkaloid production in E+ *S. phoenix* and perennial ryegrass, *Lolium perenne* L. Thus, different host-endophyte combinations appear to respond differently to sustained cutting in terms of ergot alkaloid production.

Relatively constant ergovaline production that does not diminish under intense grazing could have a synergistic effect with periodic starvation, thereby influencing the timing of the sheep crashes. The ergovaline levels we observed in cut plants (roughly 100 parts per billion), although not high enough to produce clinical disease in sheep (Tor-Agbidye et al. 2001), are high enough to have subclinical effects such as reduced weight gain or milk production (Cornell et al. 1990) that could exacerbate the effects of starvation. Furthermore, ergovaline is fat-soluble and may bioaccumulate in fatty tissues over time (Klotz 2015). The release of the alkaloid during periods of weight loss can cause toxicosis symptoms to appear even after exposure to ergovaline in the diet has ended (Reed et al. 2005). Autopsies have shown that sheep dying during crashes have severely depleted fat reserves (Gulland 1992).

Our greenhouse experiment did not account for the effects of animal saliva, which can reduce ergovaline levels in some E+ red fescue populations (Tanentzap et al. 2014). Further investigation of the St. Kilda system should explore this facet of the sheep-endophyte interaction.

Our focus in this study has been on the effects of grazing pressure on forage quantity and endophyte-mediated toxicity. Other factors, such as forage nutrient content (e.g., Hik and Jefferies 1990; Stewart et al. 2006) and plant-manufactured defenses (e.g., silica; Vicari and Bazely 1993) can vary with grazing pressure, may themselves interact with endophyte infection (Li et al. 2014), and may play a role in determining herbivore carrying capacity. Further work is needed to explore the role these factors might play in feedback regulation of sheep dynamics.

### Conclusion

Our hypothesis that productivity and relative growth rate are negatively correlated with sheep density was supported: at high density, we observed a disproportionately large drop in food availability that likely contributed to overcompensatory sheep mortality and may be a factor in the long-term resilience of the vegetation. Endophyte hyphal loads tracked sheep numbers in grazed plots, suggesting that endophytes could also play a role in feedback regulation of the sheep population, but this requires further investigation. Future work should explore the interactive role that endophytes and food limitation may play in grassland systems grazed by vertebrate herbivores.

## Data Availability

The datasets generated during and/or analyzed during the current study are available in YorkSpace, the institutional data repository of York University: https://yorkspace.library.yorku.ca/xmlui/handle/10315/34946 A metadata file to accompany the datasets is available at: https://yorkspace.library.yorku.ca/xmlui/handle/10315/34945

## Footnotes

1 Throughout this paper, the term “overcompensatory mortality” refers to sheep mortality during population crashes. The terms “overcompensation,” “partial compensation,” and “no compensation” refer to plant growth responses to herbivory sensu Belsky 1986.
